# Intracellular calcium imaging for agonist screening

**DOI:** 10.52601/bpr.2024.240026

**Published:** 2025-06-30

**Authors:** Haojie Wang, Bo Yang, Liangzhu Mo, Hua-Qian Yang

**Affiliations:** 1 Cyrus Tang Medical Institute, Soochow University, Suzhou 215123, Jiangsu, China

**Keywords:** Calcium imaging, Calcium homeostasis modulator 1, Fluo-8, jGCaMP8m

## Abstract

Calcium ions are involved in the regulation of a wide range of physiological activities such as nerve conduction, muscle contraction, cell division and gene expression through local calcium transients and global calcium oscillations. Calcium homeostasis modulator 1 (CALHM1) is a novel plasma membrane large-pore ion channel mediating calcium influx. Therefore, screening for novel CALHM1 agonists or antagonists is very important for the research of physiological and pathological processes and the development of therapeutic drugs for related diseases. In this protocol, we presented the detection of real-time calcium dynamics in transiently transfected cell lines and primary cells with genetically encoded calcium indicators or calcium indicator dyes, respectively. A comprehensive step-by-step approach has been outlined for drug screening and validation to offer a valuable guide for utilizing calcium imaging to investigate calcium-related physiological processes.

## INTRODUCTION

In biological processes, Ca^2+^ regulates numerous physiological activities, including neural conduction, muscle contraction, cell division and differentiation, cell proliferation and apoptosis, cellular energy metabolism, gene expression and regulation (Clapham 2007). Rapid increases of Ca^2+^ concentration in subcellular regions regulate rapid responses, while global changes in Ca^2+^ concentration, such as calcium oscillations, regulate slower responses. Two processes control intracellular Ca^2+^ concentrations: the exchange with extracellular Ca^2+^ and the release or reabsorption of Ca^2+^ from the endoplasmic reticulum (Berridge *et al.* 2003). Calcium imaging technology employs calcium indicators to visualize Ca^2+^ ions, enabling the observation of changes in calcium ion concentrations within cells.

Over fifty years ago, Ridgway introduced the calcium-sensitive bioluminescent protein aequorin into muscle cells, successfully recording calcium transients within muscle fibers (Ridgway and Ashley 1967). Since then, calcium indicators have rapidly evolved and can be broadly categorized into two main types: calcium indicator dyes and genetically encoded calcium indicators (GECIs).

Calcium indicator dyes can be loaded into cells quickly but cannot specifically target regions within the cells (Paredes *et al.* 2008). Commonly used chemical indicators are based on BAPTA (1,2-bis(o-aminophenoxy)ethane-N,N,N',N'-tetraacetic acid), which specifically chelate Ca^2+^ over Mg^2+^. High-affinity chemical calcium indicators include Fluo-3, Fluo-4, Fluo-8, Fura-2, Indo-1, Rhod-2, and Oregon Green 488 BAPTA-1 (Hurley *et al.* 1992; Lock *et al.* 2015; Paredes *et al.* 2008). These dyes are typically made with acetoxymethyl (AM) esters to facilitate their loading and trapping into cells.

GECIs can be further divided into single fluorescent protein-based GECIs, FRET-based GECIs composed of pairs of fluorescent proteins, bioluminescent GECIs and hybrid calcium indicators. The single fluorescent protein-based GECIs mainly include Pericams, GCaMPs, and Camgaroos. A typical GCaMP family consists of green fluorescent protein (GFP), calmodulin (CaM), and the M13 domain of myosin light chain kinase (Chen *et al.* 2013). When CaM in GCaMP binds to Ca^2+^, its conformation changes, interacting with M13, which alters the conformation of GFP and enhances its fluorescence (Nakai *et al.* 2001). FRET-type GECIs utilize pairs of fluorescent proteins to detect calcium signals through Förster Resonance Energy Transfer (FRET) and are primarily used to study conformational changes and interactions of biological macromolecules, such as TN-XL, Cameleons, and D3cpV (Zhang and Looger 2024). GECIs based on bioluminescent proteins such as aequorin, allow for the detection of calcium signaling without requiring external light sources. The first bioluminescent Ca^2+^ indicator, aequorin, was purified from the jellyfish *Aequorea victoria*, and now recombinant aequorin has become a commonly used probe for measuring intracellular Ca^2+^ (Grienberger and Konnerth 2012). Hybrid calcium indicators typically consist of a protein that specifically binds calcium ions and a chemical fluorophore. While the protein does not emit light on its own, binding to calcium ions induces conformational changes that affect the emission of the fluorophores (Shcherbakova *et al.* 2021).

Recent studies indicated that calcium homeostasis modulator 1 (CALHM1) contributes to Alzheimer's disease by regulating the excitability of neurons in the cerebral cortex (Dreses *et al.* 2008). Cyro-EM structures revealed that CALHM1 forms octamers and generates a large channel pore on the cell membrane enabling calcium entry (Ren *et al.* 2020). So far, it is only known that ruthenium red and some metal ions can inhibit CALHM1 (Syrjänen *et al.* 2023). A specific activator or inhibitor is needed to investigate the physiological and pathological role of CALHM1. Therefore, this protocol aims to do drug screening with calcium imaging.

## PRONS AND CONS

Calcium indicator dyes exhibit superior photostability and lower molecular weight, yet they fall short in terms of subcellular localization precision and have limited intracellular retention times (Paredes *et al.* 2008). Conversely, GECIs offer stronger signals and more precise subcellular localization through fusion with signaling peptides, enabling them to persist stably within cells for extended periods. However, a notable drawback of these indicators is their susceptibility to pH fluctuations, which can affect fluorescence intensity (Mollinedo-Gajate *et al.* 2019).

In this protocol, we employed Fluo-8 and jGCaMP8m as representative organic dye indicators and genetically encoded calcium indicators, respectively. These indicators are widely utilized due to their single-wavelength characteristics, with excitation peaks around 490 nm and emission peaks around 520 nm, which align with the most commonly available light sources in fluorescent microscopes. The Fluo series, which includes Fluo-3, Fluo-4, Fluo-5F, Fluo-5N, Fluo-8, and various derivatives, has seen continuous advancements, with Fluo-8 representing the latest generation, offering enhanced signal brightness (Paredes *et al.* 2008; Tsuji *et al.* 2024). Similarly, the GCaMP series has undergone iterative improvements, with GCaMP8 being the most recent derivative, characterized by higher fluorescence intensity, a darker background, and faster kinetics, resulting in an improved signal-to-noise ratio (Chen *et al.* 2022; Martín-Aragón Baudel *et al.* 2022; Masson *et al.* 2022).

## SUMMARIZED PROCEDURE

**Figure 1:**
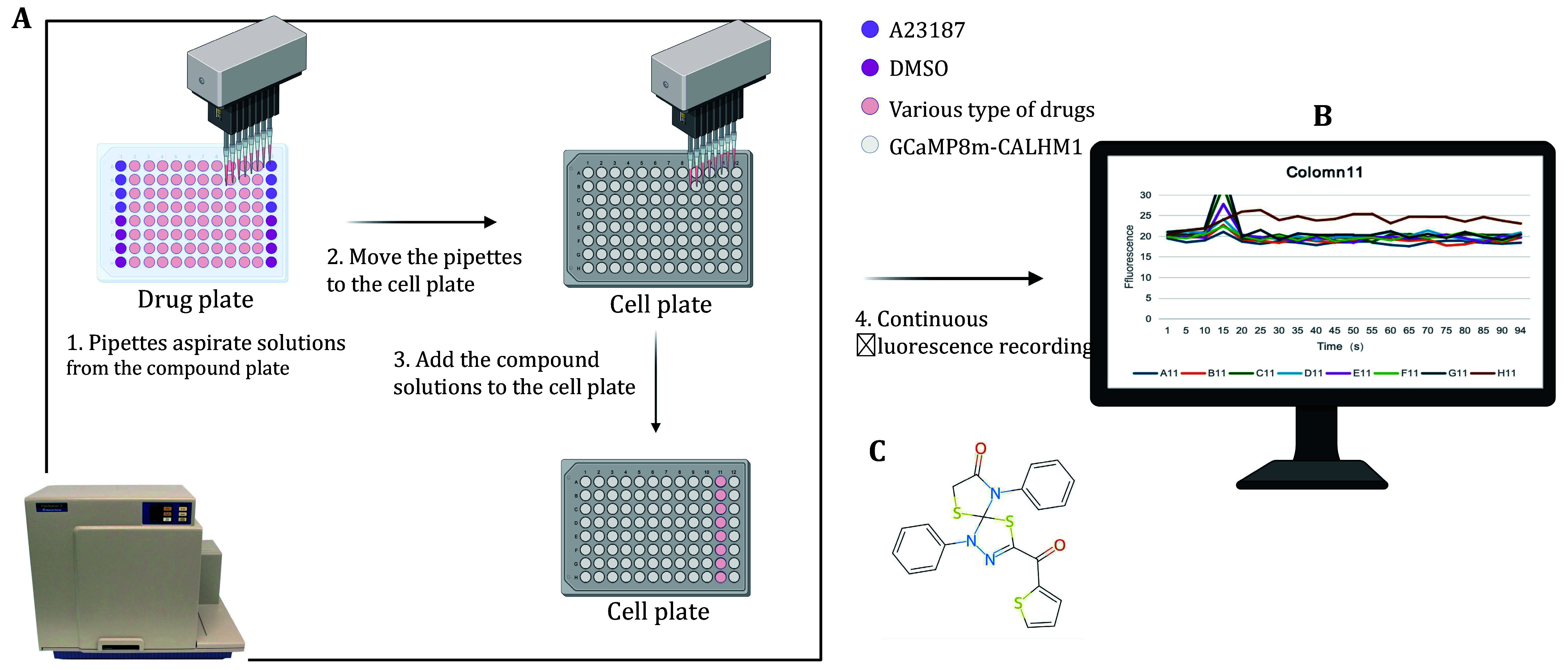
Overall flow chart of Flexstation. **A** Cultivate HEK293 cells and transfect with jGCaMP8m-CALHM1 plasmid to prepare cell plates. Take 50 µL candidate drugs and add to the cell plate. **B** Record the fluorescence intensity changes of the cells before and after adding the drugs. **C** The 2D structure of the H11 candidate drug

**Figure 2:**
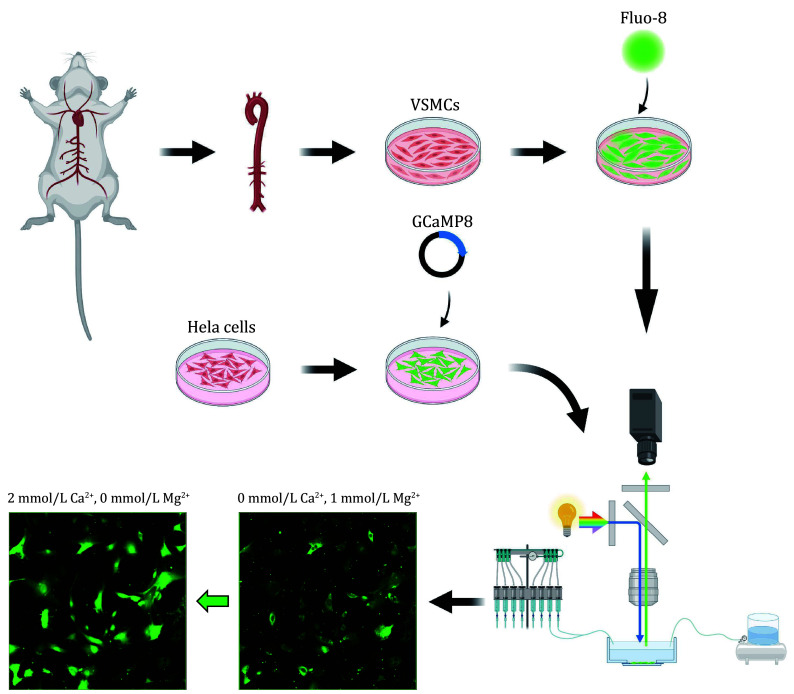
Overall flow chart of microscopic calcium imaging. Mouse vascular smooth muscle cells were isolated and incubated with Fluo-8 dye. The Hela cell line was cultured and transfected with jGCaMP8m plasmid. The changes in intracellular calcium levels were stimulated by switching from zero calcium Tyrode’s buffer (0 mmol/L Ca²⁺, 0 mmol/L Mg^2+^) to normal Tyrode’s buffer (2 mmol/L Ca²⁺, 1 mmol/L Mg^2+^) and calcium signals was detected by fluorescence microscope and photographed

This protocol aims to provide a workflow for agonist screening and validation, consisting of three steps. First, to screen for compounds that promote CALHM1 channel opening, we transfected jGCaMP8m-CALHM1 plasmid in HEK293T cells and introduced a library of more than 5000 drugs to do large-scale calcium imaging with Flexstation (Fig. 1). Second, to validate the screened candidate drugs, we describe the process of culturing HeLa cells and transfecting with the jGCaMP8m plasmid. Transfected HeLa cells are perfused with a switch of low extracellular calcium levels to high extracellular calcium levels to trigger intracellular calcium elevation and the calcium dynamics is recorded with a fluorescence microscope with much higher time and spatial resolution compared to Flexstation. Third, we provide details of calcium imaging in primary cells loaded with Fluo-8 calcium dye for further candidate drug validation. We isolate primary vascular smooth muscle cells (VSMCs) from the mouse aorta. After Fluo-8 loading, we sequentially perfused the cells with low extracellular calcium and normal extracellular calcium and recorded the changes in intracellular calcium signals (Fig. 2).

## MATERIALS AND EQUIPMENTS

### Reagents

• DMEM/High Glucose (Cytiva, Cat. #SH30243.01)

• Fetal Bovine Serum (EallBio, Cat. #03.U16001DD)

• 100× Penicillin-Streptomycin Solution (Beyotime, Cat. #C0203)

• Trypsin-EDTA Solution (Beyotime, Cat. #C0222)

• Collagenase type 2 (Worthington, Cat. #LS004176)

• Fluo-8®, AM (AAT Bioquest, Cat. #21081)

• PBS (Solarbio, Cat. #P1020)

• Cellsaving (NCM Biotech, Cat. #No: C4100)

• PolyJet™ In Vitro DNA Transfection Reagent (SignaGen, Cat. #SL100688)

• Opti-MEM (Gibco, Cat. #11058021)

• D(+)-Glucose (Hushi, Cat. #63005518)

• Magnesium chloride solution (Sigma, Cat. #BCCD5159)

• Calcium chloride solution (Sigma, Cat. #BCCD8910)

• Ethylene Glycol Tetraacetic acid (Sigma, Cat. #SLCD5533)

• Ethylenediaminetetraacetic acid (Sigma, Cat. #BCCD9612)

• HEPES (Sigma, Cat. #SLCG7609)

• Potassium chloride (Sigma, Cat. #SLCG7350)

• Sodium chloride (Sigma, Cat. #BCCD6255)

• A23187 (J&K Scientific, Cat. #380473)

• 5000 compounds library (MCE, Cat. # HY-L905)

• DMSO (SparkJade, Cat. #CS0001)

• TritonX-100 (Sigma, Cat. #9036195)

### Equipment

• Centrifuge (Kaida, TD5A)

• Electronic balance (OHAUS, PX224ZH)

• Chamber (Warner, RC-25)

• Water bath (Sapeen, DK-6D)

• Cell Incubator (Thermo, 371)

• Acidity meter (Mettler, FE28)

• Flexstation (Molecular Devices, Flexstation3)

• Miniature bench vacuum pump (Qilinbeier, GL-802b)

• Inverted fluorescence microscopy (NOVEL, NIB610-FL)

• Microscope camera (MOMENT, CMOS-MONO)

• Confocal laser scanning microscopy (Olympus, FV3000)

• Stereoscopic microscope (Nikon, SMZ745T)

### Software

• SoftMax® Pro

• Micromanager 4.0

• ImageJ

• Python Jupyter

## EXPERIMENTAL PROCEDURE

### Reagent setup

The Tyrode’s buffers with varying [Ca^2+^] and [Mg^2+^] are prepared according to the specifications in Table 1 and Table 2.

**Table 1:** Tyrode’s buffer (2 mmol/L Ca^2+^, 1 mmol/L Mg^2+^)

Components	Final concentration
CaCl_2_	2 mmol/L
MgCl_2_	1 mmol/L
NaCl	137 mmol/L
KCl	5.4 mmol/L
HEPES	10 mmol/L
Glucose	10 mmol/L

**Table 2:** Tyrode’s buffer (0 mmol/L Ca^2+^, 0 mmol/L Mg^2+^)

Components	Final concentration
EGTA	0.5 mmol/L
EDTA	0.5 mmol/L
NaCl	137 mmol/L
KCl	5.4 mmol/L
HEPES	10 mmol/L
Glucose	10 mmol/L

### Drug screening with large-scale calcium imaging in Flexstation

#### Cell resuscitation and passage

Retrieve the HEK293T cells from the liquid nitrogen tank and place them in a water bath preheated to 37°C. Shake the cryovial to accelerate thawing. After complete thawing, transfer the cells to 4 mL preheated culture medium at 37°C and centrifuge at 300*g* for 5 min. Discard the supernatant, resuspend the cell pellet in culture medium, transfer to 100 mm cell culture dishes, and culture overnight at 37°C with 5% CO_2_. Subculture the cells after two to three passages before proceeding with further experiments. Once the cell density in the 100 mm dish reaches 80%, the cells should be transferred to a 12-well plate at 20% density. Add 1 mL of PBS along the culture dish wall, aspirate after shaking, add 1 mL 0.05% trypsin-EDTA and put in the incubator for 1–2 min. Stop digestion by adding 4–5 mL culture medium, then pipette to detach the cells, transfer to a 15 mL centrifuge tube and centrifuge at 300*g* for 5 min. Discard the supernatant, add 2 mL culture medium to resuspend the cells and pipette up and down several times. Transfer 50 µL cell suspension to each well and add up to 1 mL with culture medium. Incubate overnight at 37°C with 5% CO_2_ to allow cell adhesion. It is important to recognize that the success of the experiment relies on the condition of the cells. If the cells are not in optimal condition, it is necessary to revive a new batch; otherwise, the results of the experiment may be compromised.

#### Transient transfection

Mix 1 µg jGCaMP8m-CALHM1 plasmid with 50 µL Opti-MEM medium (amount per well of the 12-well plate), vortex and centrifuge at low speed. Add 2.5 µL PolyJet DNA Transfection Reagent to 50 µL Opti-MEM and mix by inversion. Combine the two preparations and gently pipette up and down about ten times to mix. After 10 min of incubation, add to 6 wells of the 12-well plate. After 6–8 h, the transfection medium was replaced with a preheated culture medium, and a second passage was performed 24 h after transfection. Discard the original culture medium, add 400 µL PBS per well along the culture dish wall, aspirate after shaking, add 100 µL 0.05% trypsin-EDTA and put in the incubator for 1–2 min. Stop digestion by adding 400 µL culture medium, then pipette to detach the cells, and transfer the cell suspensions transfected with jGCaMP8m-CALHM1 into 15 mL centrifuge tubes. Centrifuge at 100*g* for 3 min. Discard the supernatant, add 5.5 mL culture medium to resuspend the cells and pipette up and down several times. Transfer 50 µL cell suspension transfected with jGCaMP8m-CALHM1 to a dark 96-well plate. Incubate overnight at 37°C with 5% CO_2_ to allow cell adhesion.

#### Drug testing in Flexstation

##### Preparation of candidate drugs

Under dark conditions, add candidate drugs (1 µL) to a 96-well plate (columns 2 to 11). Add 300 µL Tyrode’s buffer (2 mmol/L Ca²⁺, 1 mmol/L Mg^2+^) restored to room temperature to each well. Columns 1 and 12 are left for negative control (1 µL DMSO) and positive control (1 µL A23187), with all control wells added with 300 µL Tyrode’s buffer (2 mmol/L Ca²⁺, 1 mmol/L Mg^2+^).

##### Preparation of cell plate

Discard the original culture medium of the dark cell plate. Add 100 µL Tyrode’s buffer (2 mmol/L Ca²⁺, 1 mmol/L Mg^2+^) restored to room temperature to each well.

##### Flexstation setup

The dark transfer pipette box, candidate drugs and cell plate are prepared. Place the transfer pipette in the “tip rack”. Place the candidate drugs in the “source”. Place the cell plate in the “reading chamber”.

##### Program settings

Select the Read Mode of Fluorescence (RFUs) in “Flex” under “Settings”. The excitation wavelength is set at 488 nm and emission wavelength at 515 nm. Track the fluorescence intensity changes of jGCaMP8m after drug stimulation of the CALHM1 channel. The recording lasts for a total of 150 s. At 20 s, the machine is set to obtain 50 µL candidate drugs and added to the corresponding cell plate, resulting in a final concentration of 10 µmol/L for all the compounds. The time interval between each monitoring point is set to be 1.6 s, and a fluorescence signal trace consisting of 96 data points will be displayed (Fig. 1A).

##### Data processing

The fluorescence traces are displayed. No significant change is observed for negative controls, while a dramatic increase of fluorescence intensity occurs after addition A23187 (data not shown). The fluorescence traces with tested drugs are further evaluated. The drug at well H11 induces fluorescence intensity elevation (Fig. 1B) in jGCaMP8m-CALHM1 transfected cells. The Smiles code of H11 drug is C12(SCC(=O)-N1C1C=CC=CC=1)N(C1C=CC=CC=1)N=C(C(=O)C1=CC=CS1)S2 (Fig. 1C).

### Microscopic calcium imaging in transfected HeLa cells

#### HeLa cell transfection

The HeLa cells are cultured and transfected same as the HEK293 cells. To accommodate the requirements of statistical analysis, we typically perform plasmid transfection at a cell density of 40%, ensuring that by the time of the final experiment, the cell density reaches 80%. This approach yields a plasmid transfection efficiency of approximately 60%. Calcium imaging is performed within 24–48 h of transfection.

#### Calcium imaging with perfusion

Fix the perfusion tubes on an adjustable iron stand, and connect one side of the perfusion chamber to the outlet end of the perfusion tubes and the other side to a vacuum pump. Fill perfusion tubes with zero calcium Tyrode’s buffer (0 mmol/L Ca²⁺, 0 mmol/L Mg^2+^) or normal Tyrode’s buffer (2 mmol/L Ca²⁺, 1 mmol/L Mg^2+^) respectively, ensuring no air bubbles. Adjust the height of the perfusion tubes and the speed of the vacuum pump to achieve a steady flow rate, ensuring the outflow speed matches the removal speed of the vacuum pump. Open the micro-Manager 1.4 software, set the exposure time to 360 ms in live mode, and adjust the imaging interface accordingly. Open the multi-dimensional acquisition window, and set the number at 180 and the interval at 2 s in the time points section. The cells were incubated and pre-perfused with zero calcium Tyrode's buffer (0 mmol/L Ca^2+^, 0 mmol/L Mg^2+^) for 3 min and then perfused with normal Tyrode's buffer (2 mmol/L Ca^2+^, 1 mmol/L Mg^2+^) to induce calcium influx (Ma *et al*. 2012). The recording is saved as a video in TIFF stack format.

### Microscopic calcium imaging in vascular smooth muscle cells

#### Isolation and culture of mouse vascular smooth muscle cells

Use surgical scissors to open the mouse’s thoracic and abdominal cavities, remove the covering organs and intestines, cut the aorta and immerse it in pre-cold PBS to trim off surrounding fat. Place the aorta in digestive solution at 37°C for 10 min, wash twice with DMEM and remove the adventitia under a stereoscopic microscope. Longitudinally dissect the aorta, and scrape off the intima to obtain the media layer. Cut the aortic media into 1–2 mm² pieces, digest with 1.5 mg/mL collagenase II in DMEM at 37°C and pipette every 30 min for 1.5–2 h until the tissue fragments are completely digested. Centrifuge at 300*g* for 5 min, discard the supernatant, resuspend the pellet in VSMC culture medium (10% FBS, 1% Penicillin-Streptomycin, 89% DMEM) and plate the cells in a 33-mm culture dish (Robbins *et al.* 2014). Cultivate in a CO_2_ incubator for about five days before passage.

#### Vascular smooth muscle cell passaging

When cell confluency reaches approximately 70%, discard the culture medium and wash the cells with 1× PBS. Add 0.25% trypsin-EDTA solution to cover the bottom of the culture dish and incubate in the CO_2_ incubator for 2–3 min. Observe under a microscope until most cells detach, then add a complete culture medium to stop the digestion. Centrifuge at 300*g* for 5 min, discard the supernatant and resuspend the cells in complete culture medium. Seed the cells on sterilized 16-mm glass coverslips placed in a 24-well plate and allowed them to adhere overnight.

#### Calcium imaging with perfusion

The perfusion system is set up in the same way as for transfected HeLa cells. Dissolve 50 μg of Fluo-8 dye in 50 μL DMSO as stock solution and dilute 2 μL stock solution in 200 μL Tyrode’s buffer (2 mmol/L Ca²⁺, 1 mmol/L Mg^2+^) as working solution. Remove the VSMC culture medium, wash once with Tyrode’s buffer (2 mmol/L Ca²⁺, 1 mmol/L Mg^2+^) and add the Fluo-8 working solution to cover the cells completely. Incubate at 37°C for 10 min, remove the working solution and wash once with Tyrode’s buffer (2 mmol/L Ca²⁺, 1 mmol/L Mg^2+^). Before calcium imaging, open the perfusion chamber, coat the interface with Vaseline, take the stained coverslip with VSMCs and fix it in the perfusion chamber. Incubate with Tyrode’s buffer (0 mmol/L Ca²⁺, 0 mmol/L Mg^2+^) for 5 min (Ma *et al* 2012). Ensure the chamber is securely fixed to prevent leakage. Place the chamber under the microscope, with the excitation wavelength of 488 nm, find cells with normal morphology and appropriate fluorescence intensity and set up to capture 150 frames within 8 min. For perfusion, start with zero calcium Tyrode’s buffer (0 mmol/L Ca²⁺, 0 mmol/L Mg^2+^) and after 50 frames switch to normal Tyrode’s buffer (2 mmol/L Ca²⁺, 1 mmol/L Mg^2+^) until imaging is complete.

### Data processing for microscopic calcium imaging

#### Region of interest selection in ImageJ

**Figure 3:**
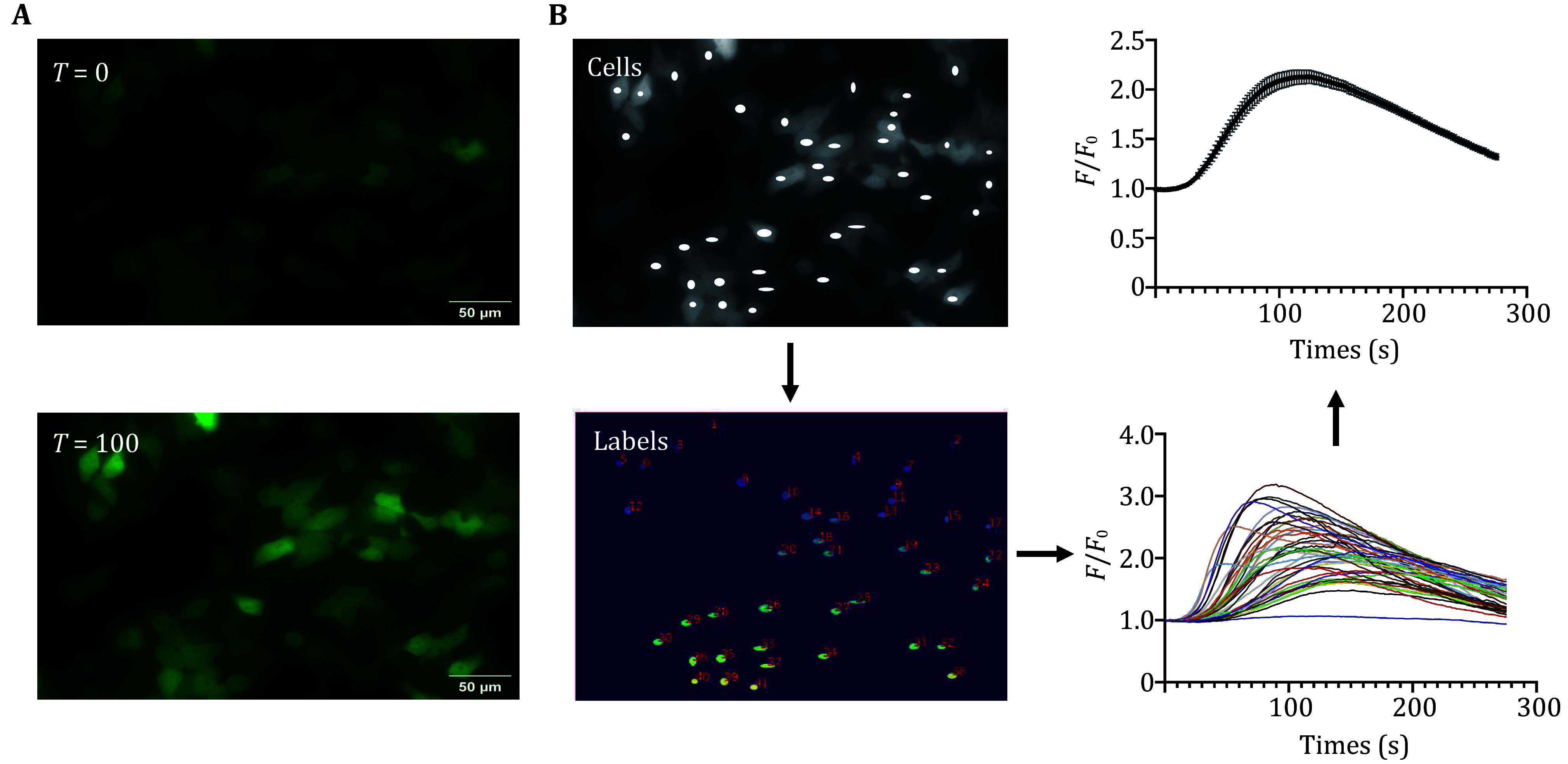
Calcium imaging data analysis with Python program. **A** *T* = 0 or 100 s, HeLa cells perfused with Tyrode’s buffer (2 mmol/L Ca^2+^, 1 mmol/L Mg^2+^). **B** The diagram “Cells” and the program outputs “labels” labeled with cell numbers. The CSV file “outputs” contains the fluorescence intensity of each labeled cell, which can be summed up to obtain the overall “Fluorescence intensity change curve”

Open the recorded TIFF video in ImageJ, select an image with all cells with high fluorescence intensity after perfusion in Tyrode's buffer (2 mmol/L Ca^2+^, 1 mmol/L Mg^2+^) and save it as a separate TIFF file. Avoiding the nuclear region, circle the region where each cell cytoplasm is located, go to Analysis/Tools/ROI Manager/Add and fill to overexposure and save it as “Cells” (Fig. 3). Circle an area without cells, overexpose in the same way and save it as “Backgrounds”.

#### Python Jupyter data processing

Execute a home-built Python program (provided in supplement materials) with Python Jupyter. It will generate the “Labels” image showing the cell numbers (Fig. 3) and a detailed CSV file named “output” containing the fluorescence intensity data points for each cell.

## CONCLUSION

This protocol outlines a systematic approach for agonist screening and validation with calcium imaging in transfected cell lines and primary cells. We used Flexstation to perform calcium imaging with jGCaMP8m to preliminarily screen for CALHM1 channel agonists from a drug library with more than 5000 drugs. To validate the potential hits, we recorded intracellular Ca²⁺ change with a fluorescence microscope in HeLa cells transfected with jGCaMP8m, which has much higher temporal and spatial resolution compared to Flexstation. Finally, we provide detailed steps for calcium imaging with Fluo-8 calcium dye in VSMCs to verify the role of agonists in primary cells. Screening with Flexstation found a potential candidate hit, but further validation is still needed. In summary, this protocol provides a detailed workflow for agonist screening and validation, including drugs library screening and further validation in cell lines and primary cells, which provides a powerful tool to explore the nature of calcium channels and the mechanism of their involvement in physiological activities,and screen for novel small molecules with potential for the treatment of related diseases.

## Conflict of interest

Haojie Wang, Bo Yang, Liangzhu Mo and Hua-Qian Yang declare that they have no conflict of interest.
